# Functionalised Mineral Materials in Environmental and Civil Engineering, Ceramics, Foundries and Metals

**DOI:** 10.3390/ma15228107

**Published:** 2022-11-16

**Authors:** Tomasz Bajda

**Affiliations:** Faculty of Geology, Geophysics and Environmental Protection, AGH University of Science and Technology in Krakow, al. A. Mickiewicza 30, 30-059 Krakow, Poland; bajda@agh.edu.pl

There has been increasing interest in functionalised mineral materials in terms of both scientific research and the development of the world economy. This arises from the growing interest in clean manufacturing technologies that use mineral sorbents, and is related to current issues in environmental and civil engineering, which are receiving significant attention both in the European Union and in international legislation. The price paid for technical progress and economic development in the 20th century was environmental pollution. In the 21st century, modern approaches based on functionalised mineral material technologies are an opportunity for world economic development and for increased industrial competitiveness in global markets while improving environmental conditions. These issues address the challenges faced by industry in protecting the environment, and in the rational use of natural resources in the creation of modern economies and in the development of industry. From the point of view of the world economy, it is important for researchers to create innovative production technologies. To meet these goals, close cooperation between science and industry is needed; thus, studies on the properties and uses of functionalised mineral materials are important.

The great focus placed on functionalised mineral materials by modern science and industry is due to their particular properties, knowledge of which interdisciplinary. The interaction of sorbents with organic and inorganic substances, and the use of mineral materials in ceramics, foundries and metals, are the subject of many publications, patents and industrial practices. These effects are fuelled by technological progress and growing demand for increasingly efficient and cost-effective technologies. Moreover, they enhance the capacity of researchers to determine the mineral and chemical composition of mineral materials, as well as their structure and texture. The second group of reasons for the developing interest in functionalised mineral materials is the progressive threat to and degradation of the environment, which demands new means of reducing the impact of industry.

Functional minerals are materials inspired by geological systems originating from Earth’s several billion-year-long history. Each of them has a unique chemical composition and structure that determine its properties and possible functions. Natural, synthetic and anthropogenic minerals, both in their original and modified forms, serve as agents in environmental and industrial applications. The functions of mineral materials include: cation and anion exchange, sorption, immobilisation, energy storage, use as catalysts and photo-activity. Layered clay minerals, zeolites and zeolite-like structures, layered double hydroxides (LDHs), are particularly suited to such functions. Modifications of minerals to obtain functional mineral materials include: surface modification, functional loading, intercalation, grafting, doping and structure reformation. 

This Special Issue, in which we will discuss topics from interdisciplinary studies, aims to highlight the current top trends in the innovative functionalisation techniques of mineral materials. Additionally, reports on unique properties of functionalised materials and their expected applications are presented.

The mineralogical and chemical characteristics of Ukrainian volcanic tuffs from the Khmelnytskyi region were determined in [1]. Their potential for use in construction and environmental technologies was assessed, with particular emphasis on their use as sorbents. Understanding the microstructure of geomaterials such as rocks is fundamental to assessing their performance and deciphering their geological history. The authors of [2] present a semi-automated statistical protocol for comprehensive three-dimensional characterisation of the microstructure of granular materials, including the clustering of grains and the description of their chemical composition, size, shape and spatial properties using 44 unique parameters. The methodological approach can be applied to any other rock type and allows microstructural trends to be tracked in the grain system. The author of [3] presents a comprehensive review of the methods used to produce foamed geopolymers and the best parameters obtained, as well as a summary of the most important information reported in the scientific literature. Moreover, they present the results of a critical analysis of the feasibility of implementing this technology for mass use. In addition, the problems and limitations most commonly encountered in the implementation of geopolymer technology are discussed. Lead–zinc sulphide leaching residue (LRT) is the only residue resulting from the leaching recovery process; it is typically a hazardous material due to its high heavy metal content and acidity. Due to the high production of LRT and the fact that its main components are Ca, Si and Al, the preparation of building materials from LRT has been investigated [4]. LRT can be used as a substitute for Portland cement, enabling more sustainable concrete production and improving the environmental performance of LRT.

The main objective of [5] was to synthesise polyurethane composites with aluminosilicates showing antimicrobial properties. The surface and antimicrobial efficacy of hybrid polyurethane films functionalised with Saponite and Phloxine B were characterised. The new method for synthesising composite materials presented in this work reveals new possibilities for the targeted modification of polymers. Such modified polymers can be useful in various applications where unique surface properties are required, for example, for materials used in medicine. With Cu_2_MnSnS_4_ (CMTS) acknowledged as an alternative to traditional semiconductors, two different sources of sulphur have been successfully used to synthesise CMTS particles via solvothermal methods [6]. The experiments show that the type of sulphur precursor used plays an important role in shaping the morphology and crystal structure of CMTS particles.

Iron-Based Water Treatment Residuals (WTRs) are an extremely interesting material, formed as a result of de-ironing and de-manganising drinking water [7]. The high content of amorphous and weakly crystalline iron hydroxides and oxyhydroxides result in a high specific surface area, which, in turn, result in excellent sorption properties in WTRs. 

The aim of the [8] was to evaluate the effect of charcoal after cavitation on the chemical and biochemical properties of soil. The cavitated charcoal used in the study showed a significant positive effect on the amount of *S. saccharatum* (L.) biomass, and its application significantly reduced the heavy metal content of *S. saccharatum* (L.) biomass. Based on data reported in the literature, Ref. [9] presents a systematic and comparative review of the uses and comparisons of pesticide adsorbents. The paper summarises information gathered from various studies, including the types of adsorbents and pesticides used, and the experimental conditions and results of each study. The studies analysed are laboratory-based and show the potential advantages of treating pesticide-containing waters using functionalised and non-functionalised synthetic zeolites and mesoporous silica materials. In general, the functionalised materials show better removal efficiency for various pesticides than conventional materials. It is hoped that the results of this review will help researchers develop an effective strategy to reduce pesticides in wastewater.

## Data Availability

Not applicable.
